# Modified live vaccine strains of porcine reproductive and respiratory syndrome virus cause immune system dysregulation similar to wild strains

**DOI:** 10.3389/fimmu.2023.1292381

**Published:** 2024-01-12

**Authors:** Katerina Stepanova, Miroslav Toman, Jana Sinkorova, Simon Sinkora, Sarka Pfeiferova, Helena Kupcova Skalnikova, Salim Abuhajiar, Romana Moutelikova, Jiri Salat, Hana Stepanova, Katerina Nechvatalova, Lenka Leva, Petra Hermanova, Mirka Kratochvilova, Blanka Dusankova, Marek Sinkora, Vratislav Horak, Tomas Hudcovic, John E. Butler, Marek Sinkora

**Affiliations:** ^1^ Laboratory of Gnotobiology, Institute of Microbiology, Czech Academy of Sciences, Novy Hradek, Czechia; ^2^ Department of Infectious Diseases and Preventive Medicine, Veterinary Research Institute, Brno, Czechia; ^3^ Laboratory of Applied Proteome Analyses and Research Center PIGMOD (Pig Models of Diseases), Institute of Animal Physiology and Genetics, Czech Academy of Sciences, Libechov, Czechia; ^4^ Institute of Biochemistry and Experimental Oncology, First Faculty of Medicine, Charles University, Prague, Czechia; ^5^ Department of Cell Biology, Faculty of Science, Charles University, Prague, Czechia; ^6^ Department of Microbiology and Immunology, Carver College of Medicine, University of Iowa, Iowa City, IA, United States

**Keywords:** Porcine respiratory and reproductive syndrome virus, thymocytes, T-cell precursors, T lymphocytes, B lymphocytes, animals

## Abstract

**Introduction:**

Porcine reproductive and respiratory syndrome virus (PRRSV) emerged about 30 years ago and continues to cause major economic losses in the pork industry. The lack of effective modified live vaccines (MLV) allows the pandemic to continue.

**Background and objective:**

We have previously shown that wild strains of PRRSV affect the nascent T cell repertoire in the thymus, deplete T cell clones recognizing viral epitopes essential for neutralization, while triggering a chronic, robust, but ineffective antibody response. Therefore, we hypothesized that the current MLV are inappropriate because they cause similar damage and fail to prevent viral-induced dysregulation of adaptive immunity.

**Methods:**

We tested three MLV strains to demonstrate that all have a comparable negative effect on thymocytes *in vitro*. Further *in vivo* studies compared the development of T cells in the thymus, peripheral lymphocytes, and antibody production in young piglets. These three MLV strains were used in a mixture to determine whether at least some of them behave similarly to the wild virus type 1 or type 2.

**Results:**

Both the wild and MLV strains cause the same immune dysregulations. These include depletion of T-cell precursors, alteration of the TCR repertoire, necrobiosis at corticomedullary junctions, low body weight gain, decreased thymic cellularity, lack of virus-neutralizing antibodies, and production of non-neutralizing anti-PRRSV antibodies of different isotypes.

**Discussion and conclusion:**

The results may explain why the use of current MLV in young animals may be ineffective and why their use may be potentially dangerous. Therefore, alternative vaccines, such as subunit or mRNA vaccines or improved MLV, are needed to control the PRRSV pandemic.

## 1 Introduction

Many childhood diseases can be traced to events during fetal and neonatal development, a period often called the critical developmental window. During this period, many organ systems complete their development, including the adaptive immune system. Viruses are involved in many of these diseases, and new ones continue to emerge. In swine, an arterivirus called Porcine Reproductive and Respiratory Syndrome Virus (PRRSV) infects the thymus, can cause thymic atrophy, alter thymocyte development and dysregulate T cell development (1). PRRSV is responsible for a pandemic, which is a very feared, frustrating, and costly infection for farmers. Losses due to abortions and stillbirths, low weight gain, and suffering from secondary infections are by far the greatest economic problems, making PRRSV a major threat to pig health and global pork production (2).

The strategy to control most viral diseases in neonates is to use vaccines. For PRRSV, inactivated vaccines have been largely unavailable in many countries since 2005 due to their low efficacy, and recombinant (DNA, subunits, and virus-vectored) appear to be far less effective than modified live vaccines (MLV), which are also far from perfect but are the most commonly used (3). Vaccination with MLV can stabilize the herd to some degree, but success is inconsistent, PRRSV frequently reappears, and cannot be eradicated. Given the etiology of PRRSV and our recent finding how PRRSV disorganizes neonatal thymic development and consequently humoral immune responses (1), it was important to determine if MLV behave differently than wild-type viruses, particularly in young animals. Indeed, previous results show that older animals that were not previously infected as piglets can produce effective VN antibodies (4–6), suggesting that this dysregulation does not occur in animals never exposed to the virus as neonates. For this reason, in this work we used three MLV and two wild strains and measured various parameters of thymocyte development, peripheral T and B cells, and stimulation of anti-PRRSV and virus-neutralizing antibodies (VN-Abs) in piglets vaccinated/infected at 5 weeks of age. The data show that at least some MLV behave in the same dysregulatory manner in young piglets as wild PRRSV. Since the economic impact of PRRSV mainly affects piglets during the critical developmental window, alternative strategies or vaccines that allow proper thymocyte and T cell development are needed to combat this viral disease. Lessons learned with PRRSV can be useful in combating other emerging viruses that interfere with the proper development and maturation of the adaptive immune system in neonates.

To understand the effect of PRRSV on development of T cells, it is useful to mention that several subsets of CD2^+^ αβ T-lineage cells can be identified in the porcine thymus (7–10). The earliest T cell precursors are CD3^—^CD4^—^CD8^—^ triple-negative (TN), which develop into CD3^—^CD4^+^CD8^+^ double-positive (DP) precursors. After TCR rearrangement and surface TCR/CD3 expression, these DP precursors become CD3^lo^CD4^+^CD8^+^ triple-positive (TP) precursors that eventually differentiate into CD3^hi^ mature single-positive (SP) CD4^+^CD8^—^ T helper (Th) cells or CD4^—^CD8^+^ T cytotoxic (Tc) cells. Infection with PRRSV causes selective depletion of DP precursors, and despite both subsequent Th and Tc cells are affected, the decrease in Tc cells is much lower than in Th cells (1).

## 2 Materials and methods

### 2.1 Experimental animals

Large white piglets from a specific pathogen-free and PRRSV-negative herd were used. Each group consisted of six 5-week-old animals housed in a separate room in biosafety facilities. The negative status of all animals before infection was confirmed by quantitative reverse transcription PCR (RT-qPCR; see Section 2.4) and by anti-PRRSV IPMA antibody titers (samples also served as negative controls; see Section 2.5). Animals were monitored regularly and animal behavior, body temperature, and weight were recorded, and blood samples and oral swabs were collected at various times during the study. Two piglets from each experimental group were sacrificed under general anesthesia at 10, 14, and 17 days post infection (dpi). The data for the cultivation experiments were supplemented and confirmed by other uninfected and age-matched animals from other experiments. Some data from older animals (large white 3-year-old adult sows from a PRRSV-free herd) infected and vaccinated according to the same protocol as the piglets were used for comparison. All animal experiments were approved by the Expert Commission for Animal Welfare of the Ministry of Agriculture of the Czech Republic according to the guidelines of the Animal Welfare Act.

### 2.2 Infections and vaccination

The Lelystad strain as a prototype of PRRSV type 1 infection and American strain VR2332 as a prototype of PRRSV type 2 infection were used as wild strains. Both viruses were propagated on the MARC-145 cell line in supplemented DMEM, and virus concentrations were determined by plaque assay. Commercial MLV strains include (1) Porcilis (Intervet International), (2) Ingelvac PRRSFlex EU (Boehringer Ingelheim) and (3) Unistrain (Hipra). These were selected based on phylogenetic tree analysis (11), with Porcilis closely related to the wild Lelystad strain, while Ingelvac is closely related to the wild VR-2332 strain. Unistrain is derived from the PRRSV type 1 strain but unrelated to Lelystad. Test piglets in each group were intramuscularly infected with a 1 ml dose of wild PRRSV (1 × 10^6^ PFU) or the recommended dosage for MLV. Our plaque assay tests resulted in the following dosages: Porcilis (2.94 x 10^5^ PFU), Ingelvac PRRSFlex EU (4.94 x 10^4^ PFU), and Unistrain (9.28 x 10^4^ PFU). Intramuscular inoculation was preferred over intranasal inoculation because it is simpler, more uniform, and has the same course of infection (12).

### 2.3 Source of samples, tissues and preparation of cell suspensions

Sera from clotted blood and fluids from oral mucosal swabs were processed as previously described (5, 13) and used for further virological and serological testing. Cell suspensions were also prepared as previously described (14–16) and used for flow cytometry and molecular analysis. Briefly, solid tissues were teased apart with forceps and then passed through a 70 μm nylon membrane. Cells from bronchoalveolar lavage (BAL) were recovered by washing the lungs with PBS. Erythrocytes from heparinized blood and other cell suspensions were removed by hypotonic lysis (13). All cell suspensions were then washed twice in PBS, filtered again through a nylon membrane, and cell numbers were determined using an automated cell counter. Portions of the same intact tissues were also frozen in Tissue-Tek for subsequent histological studies. Heparinized blood was also analyzed for white blood cell leukograms by automated hematology analyzer.

### 2.4 RT-qPCR for viral load determination

Total nucleic acids from experimental samples were extracted using a Chemagic Viral DNA/RNA kit (PerkinElmer/Revvity chemagen Technologie) according to the manufacturer’s instructions for manual use. The RNA was eluted in 60 μl RNase-free water and immediately used in the one-step RT-qPCR assay using the Luna Universal Probe One-Step RT-qPCR kit (New England Biolabs) according to the manufacturer’s instructions. Primers and probes for PRRSV type 1 infection were of our own designed based on M96262.2 reference sequence: (1) PRRSV1-qF (5’-GGAAGGTCAGTTTTCAGGTTG-3’), (2) PRRSV1-qR (5’-AATTAACTTGCACCCTGACTGG-3’), and (3) PRRSV1-qPR (5’-HEX-AGTTTATGCTGCCGGTTGCTCATAC-BHQ1-3’). Primers and probes for PRRSV type 2 infection were of published or modified design (17) based on EF536003 reference sequence: (1) PRRSV2-U-F (5’-TCCAGATGCCGTTTGTGCTT-3’), (2) PRRSV2-U-R (5’-CCCAACACGAGGCTTTTYAA-3’), and (3) PRRSV2-U-P (5’-FAM-TCTGGCCCCTGCCCACCA-BHQ1-3’). Quantification of viral genome copies was based on RNA quantification standards prepared using the RiboMAX™ Large Scale RNA Production System SP6 (Promega) according to the manufacturer’s instructions. Viral concentrations were converted to 1 ml of body fluid or 1 g of tissue.

### 2.5 Immunoperoxidase monolayer assay

Antibodies capable of recognizing the virus were detected by the IPMA assay according to Wensvoort et al. (18), which was performed in a certified laboratory of the State Veterinary Institute, Jihlava, Czech Republic. MARC-lineage cells were used for virus propagation, and sheep anti-pig IgG antibodies conjugated with horseradish peroxidase were used for visualization using 3-amino-9-ethyl-carbazol.

### 2.6 Measurement of total immunoglobulin isotype concentrations (ELISA)

Total serum IgM, IgG, and IgA concentrations were determined by Sandwich ELISA as previously described (19, 20). Polyclonal goat anti-pig IgM (Bio-Rad AAI48), IgG (Bio-Rad AAI41), and IgA (Bio-Rad AAI40) antibodies were used for capturing of porcine Ig from sera. For detection, monoclonal primary antibodies for pig IgM (M160) and IgA (M1459) were used, followed by secondary goat anti-mouse polyclonal antibodies labeled by alkaline phosphatase (Merck A2179). For porcine IgG detection, polyclonal goat anti-pig IgG antibodies labeled by biotin (Merck SB6 050-08) were used, followed by ExtrAvidin labeled by alkaline phosphatase (Merck E2636). Plates were developed with pNPP liquid substrate and processed/analyzed with BioTek Synergy HTX system at 405/630nm dual readings. Inter-assay ELISA variability (coefficient of variation) was 10% for IgM, 14% for IgG, and 11% for IgA. Because absolute values were variable among piglets, data are reported as percent changes in individual animals (calculated back to 0 dpi as 100%).

### 2.7 Virus neutralization test

Titers of VN-Abs were determined from heat-inactivated sera (56°C for 30 minutes) serially diluted twofold in 96-well microplates with DMEM medium supplemented with 3% FBS containing 50 PFU PRRSV. Each sample was analyzed in two simultaneous assays with both viral strains. After incubation (60 minutes at 37°C), MARC-145 cells (3 × 10^4^) were added to each well, and the cytopathic effect was determined microscopically after 5 days of cultivation. The reciprocal value of the final serum dilution causing a 50% reduction in cytopathic effect was defined as the VN-Abs titer. Each measurement included sera with known VN-Abs titer as positive controls. The positive sera were isolated from a different experiment with old sows that were responsive to PRRSV and which had been treated similarly to the experimental samples.

### 2.8 Staining of cells, flow cytometry, and cell sorting

Cell staining was performed by indirect subisotype staining as previously described (14, 15). Briefly, multicolor staining was accomplished by incubating cells with a combination of four primary mouse anti-pig monoclonal antibodies of different subisotypes: CD2 (1038H-5-37, IgM), CD3 (PPT3, IgG1), CD4 (10.2H2, IgG2b), CD8 (76-2-11, IgG2a), CD21b (IAH-CC51, IgG2b), and Igμ-heavy chain (M160, IgG1). After incubation for 15 minutes followed by washing, mixtures of secondary goat anti-mouse Ig subclass polyclonal antibodies labeled with different fluorochromes were added to the cell pellets in appropriate combinations. After 15 minutes, the cells were washed three times and were ready for cell surface cytometry. All immunoreagents were titrated to an optimal signal-to-noise ratio and isotype-matched mouse anti-rat monoclonal antibodies were used as negative controls. Stained cells were measured on FACSCalibur or sorted on FACSAria-III flow cytometer. Electronic compensation was used to eliminate residual spectral overlaps between individual fluorochromes. FSC-area/FSC-width parameters were used to eliminate doublets. Software PC-LYSYS or FACSDiva was used for data processing and gating strategy is described in **Supplementary Material 1**.

### 2.9 Cell cultures

Cell cultures were used to study the extent of different MLV strains to cause dysregulating effect on precursor and mature thymocytes. Cultures were performed in RPMI-1640 medium supplemented with L-glutamine and 25 mM HEPES, 10% fetal bovine serum, 100U penicillin and 0.1 mg/ml streptomycin in CERTOMAT CS-20 CO_2_ incubator. The final concentration of thymocytes was always adjusted to 2x10^6^ cells/ml and cells were cultured with medium alone or 0.1 vaccination dose per ml. Cells were harvested at different time points, surface stained (see chapter 2.8.) and analyzed by flow cytometry.

### 2.10 RNA isolation and PCR amplification of T cell receptor beta-chain variable gene transcripts

Different populations containing 100-300 thousand sorted cells were dissolved in 1 μl TRI reagent per thousand cells. Samples within a particular experiment used the same number of sorted cells for total RNA isolation and cDNA preparation using random hexamer primers according to a protocol recommended by the manufacturer. The first round of PCR targeted the original cDNA preparation while the second round of PCR targeted the product from the first round. A detailed description of analyzes, addressing sample quality and recovery efficiency has been described previously (21, 22). All primers used for PCR have also been published previously (23). Specifically in this report, primers VβIV, α-Cβ1, and α-Cβ2 were used for amplification of the VβIV - VβVI superfamily, which constitutes > 50% of the TRBV repertoire (23–25).

### 2.11 Cloning and sequencing

Cloning was performed as previously described (25, 26). Briefly, PCR-amplified and purified products were reamplified with Pfu polymerase, and isolated fragments were cloned into EcoRV-digested pBluescript II SK phagemids by T4 DNA ligase blunt-end ligation. The ligation mixture was used to transform DH5α-competent cells. Clones containing DNA fragments of the expected size after XhoI/XbaI restriction digestion were sequenced using commercial Sanger sequencing. On average, 15 sequences were recovered for each cell subset, and candidate piglets were selected based on thymic destruction status (not all samples from all piglets were cloned).

### 2.12 Microscopy

Bright-field microscopy and H&E staining were used for morphological examination of Tissue-Tek-processed cryosections. Stained sections were embedded in glycerin jelly and visualized using the Olympus BX61VS microscope and VS-ASW 2.9 software. Confocal microscopy was used for fluorescence detection of CD4 and CD8 expression compared with DAPI counterstaining of nuclear DNA in tissue after fixation of 10-μm cryosections with acetone at -20°C for 10 minutes. The same staining strategy as described in section 2.8 was used, but the incubation and washing steps were extended. Processing also included initial blocking with 10% normal porcine serum. Cryosections were embedded in Mowiol medium, visualized using the Leica TCS SP5/LAS AF microsystem, and finally analyzed using ImageJ software.

### 2.13 Statistical analyses

Statistical analyses were performed using GraphPad Prism software. Error bars are plotted as SD from actual values, and statistical significance was measured by ordinary one-way ANOVA (two-way ANOVA for virus concentration) with Tukey’s multiple comparison tests. Statistically significant differences are indicated by asterisks (* P ≤ 0.05 ** P ≤ 0.01 *** P ≤ 0.001 **** P ≤ 0.0001).

## 3 Results

### 3.1 All MLV affect thymocytes comparable to wild PRRSV strains

**Figure 1:**
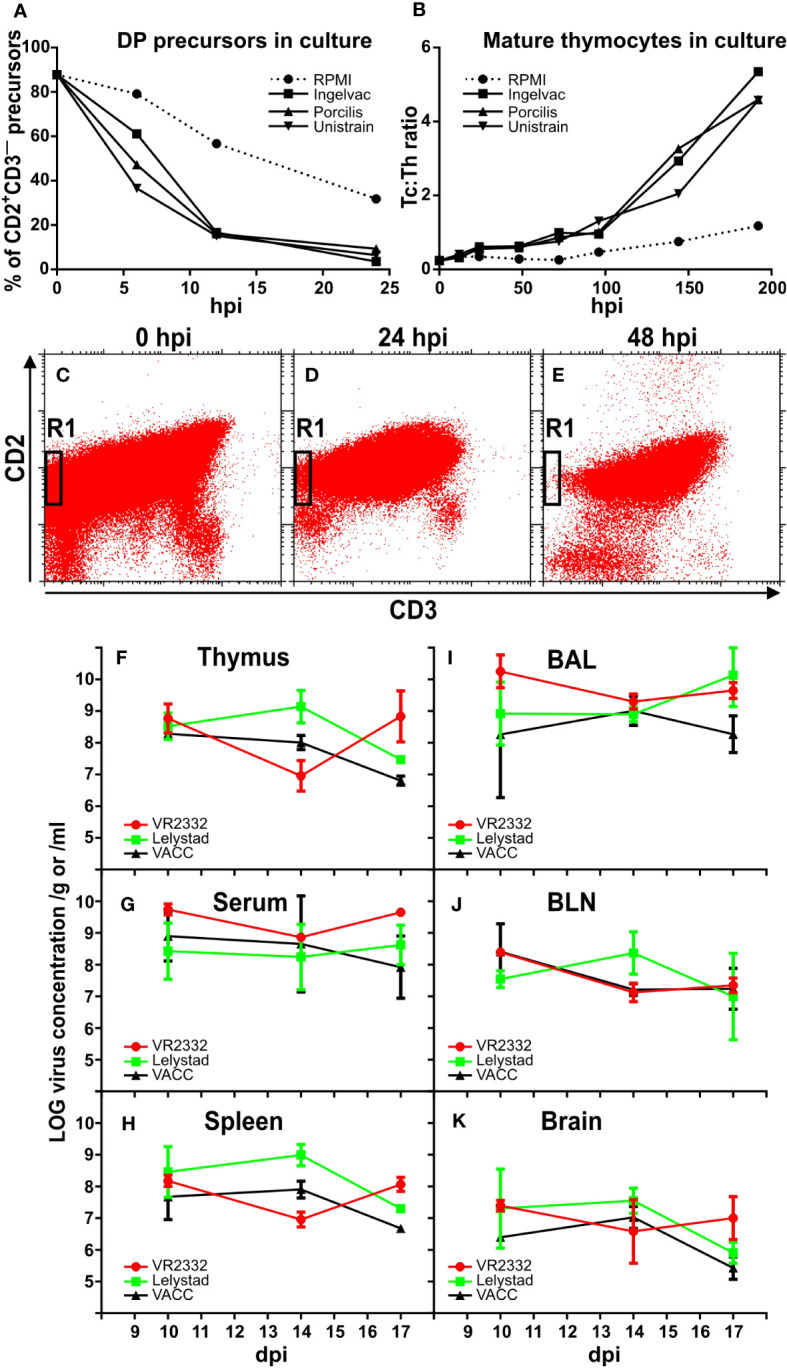
Effect of MLV on thymocytes *in vitro* and viral loads *in vivo*. Suspensions of thymocytes were cultured in parallel with medium or different MLV (Ingelvac, Pocilis, or Unistrain) for various hours (hpi). After culturing, the resulting cells were stained for CD2, CD3, CD4, and CD8 and analyzed by flow cytometry for DP precursors within CD2^+^CD3^—^ cells **(A)** and for the Tc : Th ratio between SP mature cells **(B)**. The results are representative of three independent experiments performed with different hpi. The decrease in CD2^+^CD3^—^ precursors (region R1) in MLV cultures is illustrated in representative dotplots showing the initial suspension **(C)** and the situation after 24 hpi **(D)** and 48 hpi **(E)**. In the subsequent *in vivo* experiment, MLV were used in a mixture (VACC) and compared with wild strains VR2332 and Lelystad after i.m. infection of piglets. Viral levels were monitored by RT-qPCR in various tissues **(F–K)** including bronchoalveolar lavage (BAL) and bronchial lymph nodes (BLN). No significant differences were detected in any tissue between the different PRRSV strains including VACC.

The knowledge on development of T cells in the porcine thymus mentioned at the end of the introduction was used to estimate the effect of different MLV strains on thymocytes when cultured with RPMI medium alone, or with Ingelvac only, or with Porcilis only, or with Unistrain only. Flow cytometric analyses of different subpopulations after various time points of *in vitro* culturing revealed that the proportion of DP precursors decreases much faster for all three MLV strains in comparison with medium alone (**Figure 1A**). Also in agreement with the expected effect of PRRSV was the finding of much higher ratio between Tc: Th mature thymocytes (**Figure 1B**). The demonstrative picture of vigorous precursor depletion in region R1 of cytometry dotplots is shown in **Figures 1C–E**. These data show that all three MLV strains have comparable effects on thymocytes, and therefore they were used as a mixture in all subsequent *in vivo* studies. This MLV mixture is hereafter referred to as VACC, and its effects were compared with the wild strains VR2332 and Lelystad, and with uninfected piglets (CTRL) serving as controls. Monitoring of *in vivo* infections showed that all experimental piglets were successfully infected, and that the level of detectable virus is comparable (not significantly different) in all monitored tissues (**Figures 1F–K**). The viral load was also comparable during 10-17 dpi when all animals were analyzed. For this reason, some data from these time points were processed together in further analyses as the effect was comparable. The virus of all PRRSV strains had not disappeared by 17 dpi (**Figures 1F–K**).

### 3.2 MLV also behave comparably to wild PRRSV strains in other parameters

**Figure 2:**
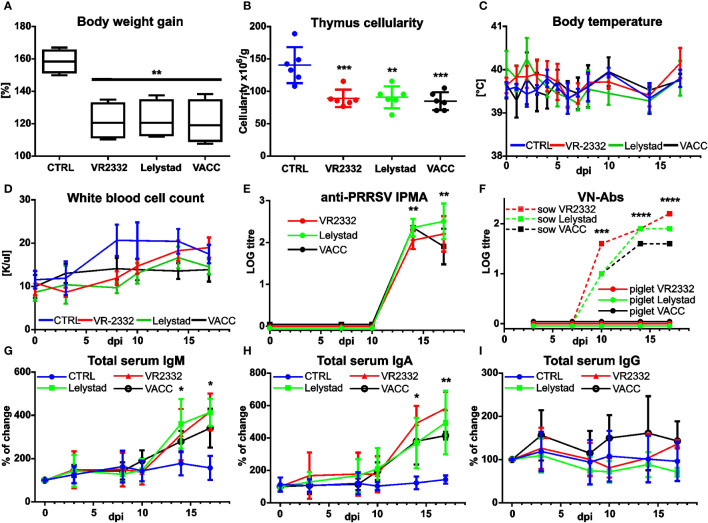
Effect of MLV and wild PRRSV strains on other infection parameters. Control piglets (CTRL) and piglets infected with wild VR2332, Lelystad and a mixture of MLV strains (VACC) were monitored throughout the experiment for their weight gain (calculated back to 0 dpi as 100%) after two weeks **(A)**. Cell numbers in the suspensions obtained from each thymus were recalculated per gram of tissue used for the preparation **(B)**. Body temperature was also recorded **(C)**. Blood collected at different time points was examined for white cell count using a hematological analyzer **(D)**. Antibodies capable of recognizing the virus were analyzed in the sera by IPMA **(E)**, but none of the piglet sera was able to neutralize the virus according to the virus neutralization test **(F)**. Note that the positive control sera (dashed lines in F) were from a different experiment using old sows. Total IgM **(G)**, IgA **(H)**, and IgG **(I)** from sera were also analyzed by ELISA. Note that data are expressed as percent changes in individual animals (calculated back to 0 dpi as 100%). Time points at which infected piglets are significantly different from CTRL **(A, B, E, G, H)** or older pigs from younger piglets **(F)** are indicated by asterisks. No significant differences were detected in any tissue between the different PRRSV strains including VACC.

Several parameters were monitored and analyzed during *in vivo* infections. Significant differences between CTRL and infection groups were observed in weight gain (**Figure 2A**) and thymus cellularity (**Figure 2B**), whereas no differences were observed in other parameters such as body temperature (**Figure 2C**), leukograms (**Figure 2D**), animal behavior, or health scores. Previous observations have shown that infection causes a production of anti-PRRSV antibodies, but these are unable to neutralize the virus (27; reviewed in 28 and 29). The same result was obtained for all PRRSV strains, including VACC (**Figures 2E, F**). The virus neutralization test included immunized old sows as positive controls (**Figure 2F**, dashed lines). PRRSV infection is also characterized by a large and unexpected increase in total IgM, IgG, and IgA levels, especially in fetal, germ-free, and very young piglets (23, 30–33). Interestingly, we found a significantly different increase in IgM (**Figure 2G**) and IgA (**Figure 2H**) but not for IgG (**Figure 2I**) for all PRRSV strains, including VACC.

### 3.3 MLV cause the same depletion of CD4^+^CD8^+^ precursors in the thymus as wild strains

**Figure 3:**
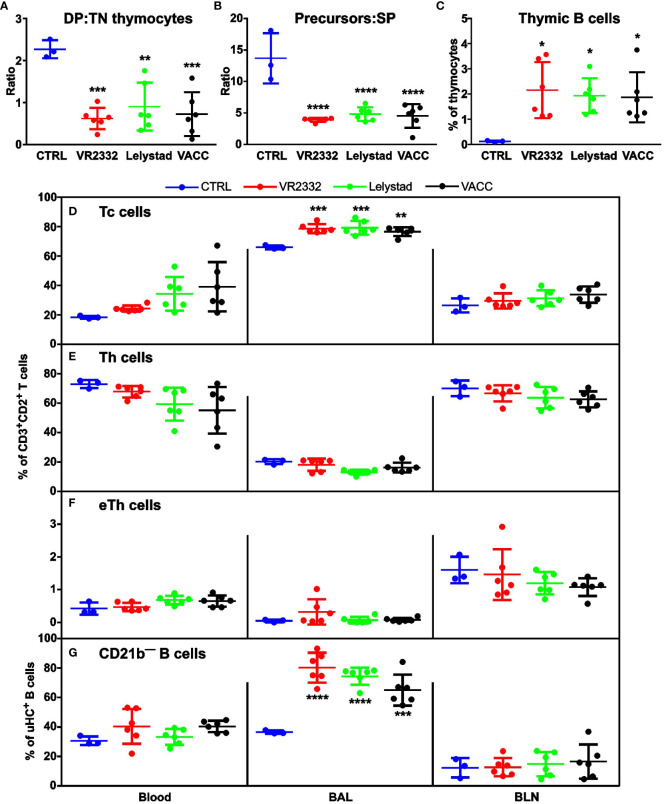
Analysis of T and B cell subpopulations in thymus and periphery. Cell suspensions from thymus, blood, BAL, and BLN were stained for CD2/CD3/CD4/CD8 and μHC (Igμ)/CD2/CD21b and examined for differences in the ratio of DP: TN thymic precursors **(A)**, in the ratio of CD2^+^CD3^—/lo^ thymic precursors: SP mature thymocytes **(B)**, and occurrence of thymic μHC^+^ B cells **(C)** within all thymocytes. Analysis of different peripheral tissues (X-axes in **(D–G)**) was performed for Tc cells **(D)**, Th cells **(E)**, and eTh cells **(F)** within CD3^+^CD2^+^ T cells. Proportion of CD21b^—^ B cells **(G)** was analyzed within μHC^+^ B cells. Data that are significantly different from CTRL are indicated by asterisks. Gating strategy is described in **Supplementary Material 1**.

*In vivo* analysis of lymphocytes also revealed several differences from CTRL, while all PRRSV strains including VACC were comparable. As mentioned in section 3.1, PRRSV infection leads to a selective depletion of DP precursors in the thymus. The same effect was observed *in vivo* and is most apparent in the ratios of DP: TN (**Figure 3A**) and precursors: SP mature T cells (**Figure 3B**). All strains used significantly differed from CTRL. An interesting feature of PRRSV infection is the relative increase in the proportion of thymic B cells because T-lineage thymocytes are depleted and thymic cellularity decreases (1), which was also found for all strains in this study (**Figure 2B**). Thymic B cells generally represent < 1% of all thymocytes (34), which is also true for CTRL animals (**Figure 3C**). On the other hand, a significant increase in the proportion of thymic B cells was observed in all infections, including VACC (**Figure 3C**). Peripheral T cells were analyzed for their CD4^—^CD8^+^ Tc subset (**Figure 3D**) and two Th subsets: CD4^+^CD8^—^ (**Figure 3E**) and CD4^+^CD8^lo^ effectors (eTh; **Figure 3F**). We found no significant differences in the effect of infection in most cases and different tissues, except for Tc cells in BAL and again for all infections (**Figure 3D**). Regarding B cells, they express CD21 in two forms, and only CD21b expression is diminished after activation (16, 35). A significant increase in the proportion of CD21b^—^ effector B cells was again observed only in BAL (**Figure 3G**). It should be noted that there were no significant differences between the different PRRSV strains in any of the lymphocyte analyses.

### 3.4 Depletion of thymocytes is visible near the corticomedullarly junctions

**Figure 4:**
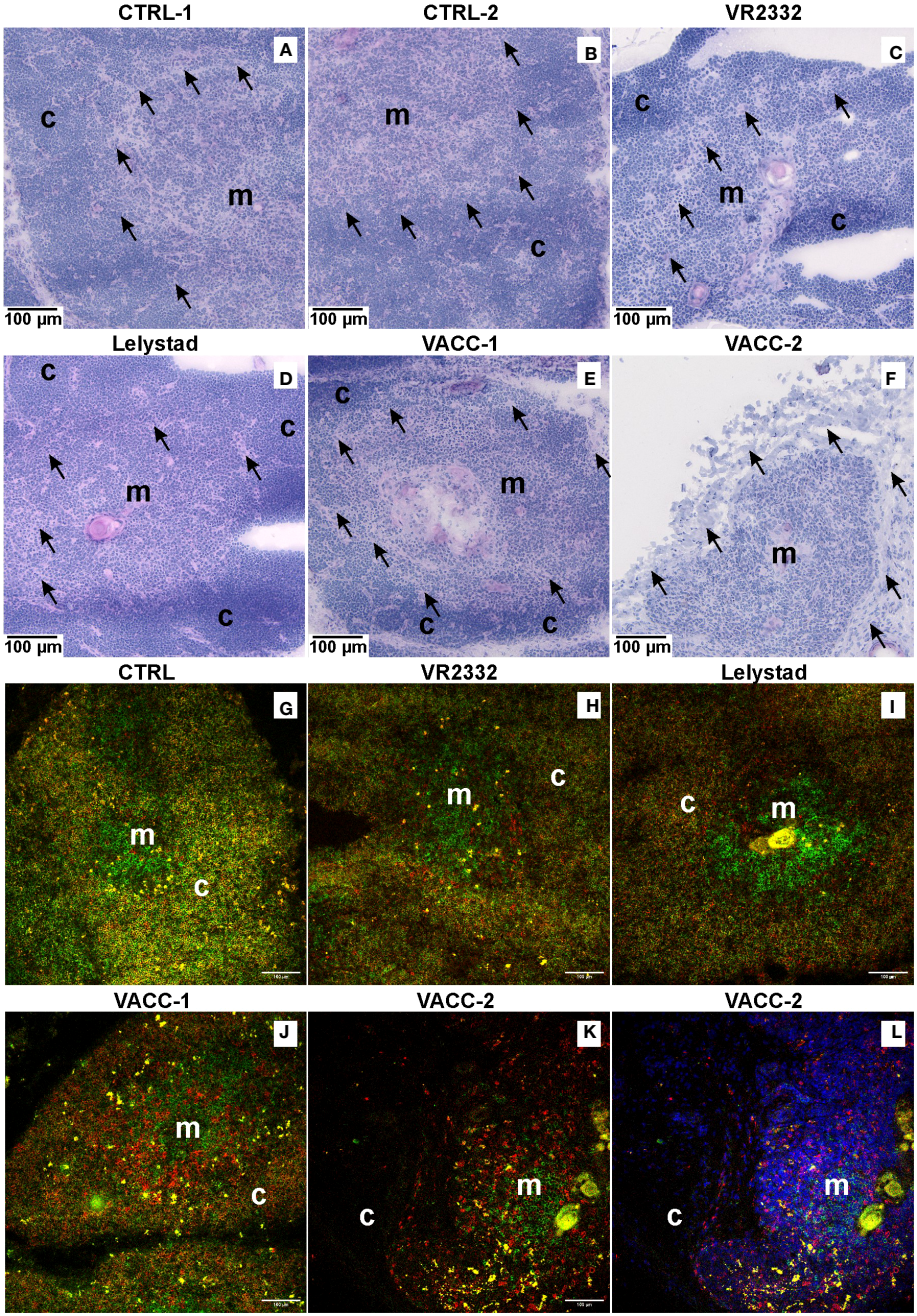
Both MLV and wild PRRSV strains cause depletion of DP precursors at the cortico-medullary junction. H&E staining was used to examine the overall thymic histology for two CTRL **(A, B)**, VR2332-infected **(C)**, Lelystad-infected **(D)**, and VACC-inoculated **(E, F)** piglets at 14 and 17 dpi. Two VACC piglets are shown to demonstrate mild **(E)** and severe **(F)** damage. Medulla (m) and cortex (c) are depicted, and arrows indicate corticomedullary junctions where marked necrobiosis is present in PRRSV-infected animals. Confocal microscopy of sections stained for CD4 (green) and CD8 (red) was used to demonstrate loss of cortical DP precursors (yellow), namely at cortico-medullary junction. CTRL animals **(G)** are compared with VR2332 **(H)**, Lelystad **(I)**, and VACC **(J)**. Also shown is severe damage in some animals **(K)** with nuclear DAPI counterstaining **(L)** demonstrating depletion of CD4 and/or CD8 positive thymocytes.

Histology of the thymus showed no pathologic changes in CTRL animals, which had a normal thymic structure with well-defined cortical and medullary regions (**Figures 4A, B**). Virus-infected animals exhibited markedly reduced cortical structure at the expense of the medulla, with evident pathologic necrobiosis in the inner cortical region near the corticomedullary junctions. This pathology was observed in all animals receiving wild VR2332 (**Figure 4C**), Lelystad (**Figure 4D**), or VACC (**Figure 4E**) strains. It is important to note that PRRSV-infected animals could be divided into two groups, and this applies also for VACC. One group shows mild thymic damage (**Figure 4E**), whereas a second group shows severe damage with almost no cortex (**Figure 4F**). In swine and other mammals, developing thymocytes migrate from the outer cortex to the corticomedullary junction, where DP precursors undergo negative selection, and only SP thymocytes enter the medulla (36, 37). To detect the disappearance of DP precursors at the corticomedullary junction, we used confocal microscopy (**Figures 4G–L**) and analyzed CD4 (green color) and CD8 (red color) expression. This approach confirmed the normal thymic structure in CTRL animals (**Figure 4G**), in which the medulla contains mainly immature CD4^+^ or CD8^+^ T cells (green and red are separated), whereas the cortex is densely packed with many DP precursors (yellow color for colocalized green and red cells). On the other hand, all three infected groups (**Figures 4H–J**) have sparse cortex with a striking reduction of yellow DP precursors. Severe thymic damage with absent cortex (**Figure 4K**) is also evident from DAPI nuclear staining (**Figure 4L**), which shows a depleted cortex with few cells negative for CD4 and/or CD8. This indicates cortical areas where only thymic stromal cells remain.

### 3.5 MLV restrict the TRBV repertoire similarly to wild strains

**Figure 5:**
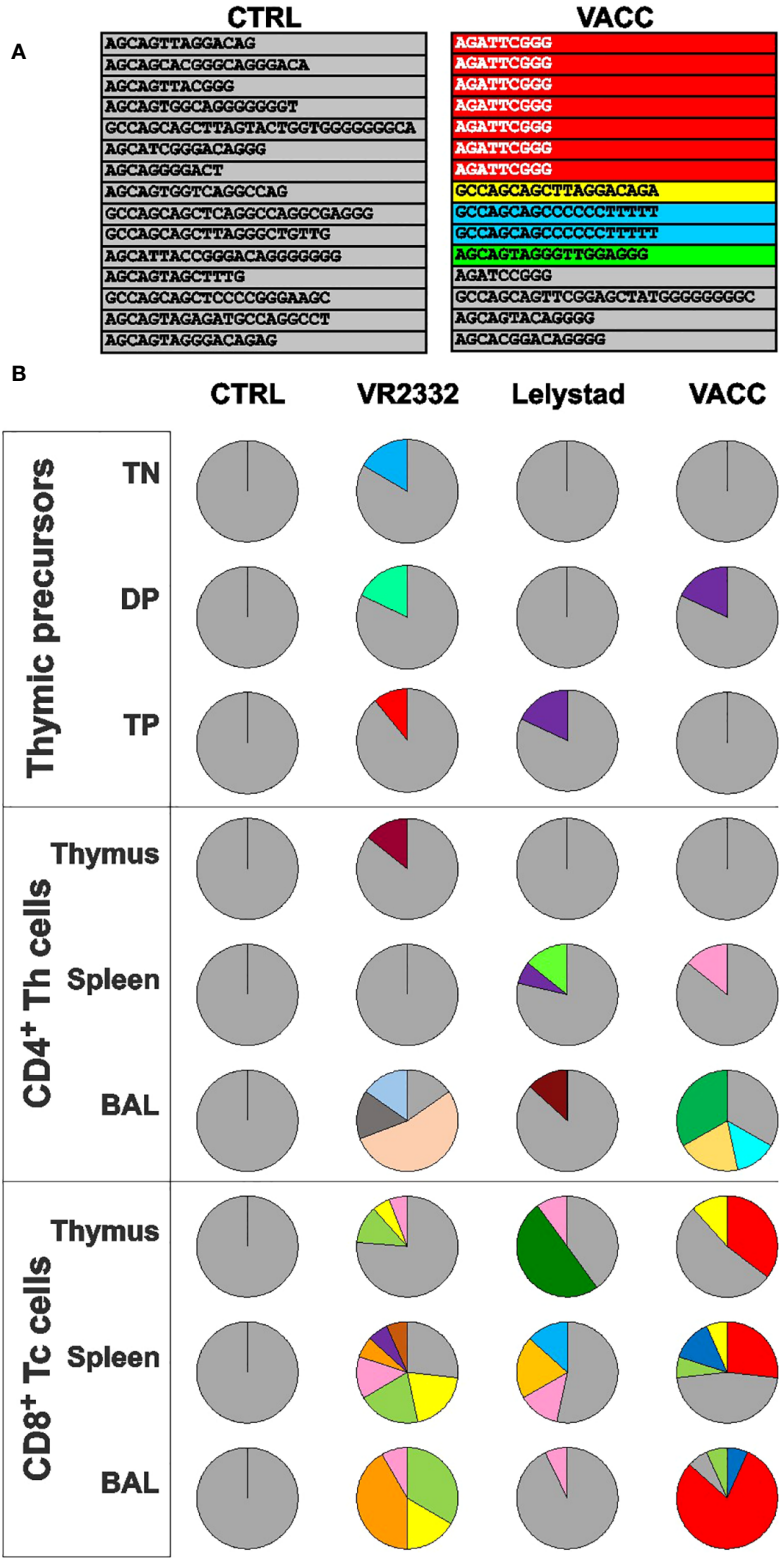
Sequence analysis of the expressed TRBV repertoire obtained from sorted T cell subpopulations. Individual T-lineage subsets from thymus and peripheral tissues were sorted by flow cytometry, and the recovered TCRβ transcripts were cloned, sequenced, and analyzed. An example of such analysis for CTRL and VACC piglets is shown in **(A)** (for simplicity, only CDR3 sequences are shown without the constant part of the TRBJ segment). All nucleotide sequences from CTRL pigs have their unique and distinct CDR3 regions (gray color). In contrary, some T cell subpopulations from infected pigs contained shared and repetitive clones with identical TRBV sequence and the same CDR3 (colors other than gray). In this particular example, some Tc cells from the VACC spleen carry colored TCR, which were also found in the thymus and/or BAL, and the clones in red and blue were also repetitive, while green and yellow were shared only. The overall results of the different experimental groups for all sorted subsets are summarized in **(B)**. Note that the data are demonstrative and the individual colors correspond to individual shared sequences within that piglet but not between different piglets. Details on all CDR3 sequences for all cell subsets can be found in **Supplementary Material 2**.

Previous findings (1) have shown that precursor depletion in the thymus leads to a restriction of the TCRβ repertoire. This is evident from the repeated and completely identical TRBV nucleotide transcripts (including the same CDR3 regions) obtained from different T cell subsets sorted by flow cytometry. The same study shows that some of the repeated clones in the thymus are the same as those in the periphery and are therefore shared. Repeated and shared clones were not observed in any of the CTRL tissues (**Figure 5A** for demonstration and **Figure 5B** for overall results), where all nucleotide sequences have their unique and distinct CDR3 regions (gray color). In PRRSV-infected animals, repeated clones (colors other than gray) are sometimes found among the precursors as their numbers and repertoire decrease (**Figure 5**, thymic precursors). However, these clones are not shared with the periphery. Note that the individual colors correspond to identical sequences within an analyzed piglet but not between different piglets. On the other hand, mature T cells from the thymus and periphery contain many repeated and shared clones. Shared clones are mainly found in CD8^+^ Tc cells but not in CD4^+^ Th cells (**Figure 5**, compare the thymus and peripheral tissues for Tc and Th cells). On the other hand, Th cells are characteristic for the occurrence of repeated clones, especially in BAL as the primary site of infection. Apparently, the shared clones are different in individual PRRSV-infected piglets. Overall, **Figure 5** demonstrates that VACC strains restrict and affect the T cell repertoire in a similar manner as wild strains. Complete details of all CDR3 sequences for all cell subsets can be found in **Supplementary Material 2**.

## 4 Discussion

This study shows that vaccination with MLV negatively affects the development and response of the adaptive immune system in the same manner as infection with wild PRRSV strains. In both groups, the viruses invade the thymus and affect the developing DP thymocytes at the corticomedullary junction where negative selection occurs (36, 37). Molecular data confirmed that the depletion is specific and affects the final thymic TCR repertoire, which is redistributed to the periphery. Since thymic infection occurs when central tolerance is still developing, PRRSV-infected piglets may tolerate the protective epitopes as self. Missing virus-specific Tc cannot efficiently kill infected cells and missing cognate Th cells cannot establish and drive the germinal center reaction and cannot help B cells to produce high-affinity VN-Abs (1, 27, 29). However, the remaining Th cells stimulate other B cells to produce antibodies with somatically non-mutated variable genes in germline configuration that are broadly specific for PRRSV epitopes (30–32, 38). The virus cannot be eliminated, and its non-neutralizing antigenic structures only lead to an amplified and ineffective immune response that cannot be terminated by antibody feedback (39). This is entirely consistent with the exaggerated antibody response against non-neutralizing nucleocapsid and non-structural epitopes compared to deficient response against neutralizing envelope epitopes (27).

Comparing the results of this work with previous studies, two major differences can be identified regardless of the PRRSV strain. The first is the finding of a less differential increase in total Ig (**Figures 2G, H**). We have previously reported tremendous increase in total Ig in fetal and germ-free piglets (23, 30–33), but the results have been criticized (29). For this reason, subsequent studies included conventional neonatal piglets to demonstrate the same effect (1). However, when older piglets are used (as in the case of the present study) the differences begin to disappear. The same is true for the second major difference, which is the finding of less apparent activation of T and B cells. While in the present study we found significant changes only in BAL (**Figures 3D, G**), previous studies with younger animals showed remarkable differences in almost all peripheral tissues except the adjacent BLN (1). Since the vast majority of other PRRSV studies have been performed with older animals, at least two effects may contribute to the differences in Ig levels and T and B cell activation that decrease with age. The first is the maturation status of the thymus. Animals may be old enough that central and peripheral tolerances are fully developed and PRRSV cannot establish host tolerance to itself. The second is related to completed colonization and associated immune system changes, which have been reported and discussed several times (20, 22, 38, 40–46). Aging animals have better immunoregulatory functions that prevent T and B cell overactivation. They also have increasing Ig levels, so the changes are less obvious. This would explain why we detect significant increase in IgM (**Figure 2G**) and IgA (**Figure 2H**), but not IgG. Studies with younger neonatal piglets showed up to a 20-fold increase in IgM and an 8-fold increase in IgA, but only a 3-fold increase in IgG (1). In any case, the results for IgM (**Figure 2G**) and IgA (**Figure 2H**) in the 5-week old piglets used in the present report still show an unceasing increase, which does not correspond to the normal primary immune response of virus-naive animals. Instead of the expected decrease in IgM levels after 14 dpi, we observed a further increase without plateau. This effect is likely related to an amplifying anti-PRRSV response against non-neutralizing epitopes that cannot be terminated by antibody feedback (39). However, the time period after which PRRSV no longer affects Ig levels and abnormal lymphocyte activation and when the thymus becomes resistant to PRRSV-induced dysregulation requires further investigation.

It is important to note that the vast majority of experiments have used animals that have never been infected with PRRSV (so-called PRRSV-free animals). Therefore, a question arises as to how secondary infection (vaccination) behaves in animals that have undergone primary infection shortly after birth versus animals that become infected later in life. Reports from the field indicate that pigs infected early in life can tolerate the virus for a very long time and in the meantime infect new waves of newborn piglets (47). This may explain why PRRSV reappears especially in large herds with many newborns and why the method to eradicate the virus is only the repopulation of entire farms. Continuous and repeated vaccination with MLV is likely problematic given the results of this report. Vaccination of animals that encountered PRRSV (or MLV) in the neonatal period and may tolerate viral epitopes is questionable. They may be virus carriers but resistant to the effects of the virus. Vaccination may also result in artificial spread of the virus throughout the herd, including very young piglets, which may remain a refuge for the virus.

PRRSV infection results in fetal abortions and increased mortality and morbidity among neonatal piglets but rarely in older animals, with exception of certain highly virulent strains (48). Thymic dysregulation decreases the ability of neonates to eliminate the virus but does not affect the development of other T cells needed for adaptive immune responses to other pathogens, as evidenced by an intact antibody response to unrelated antigens (27). This is distinct from immunodeficiency diseases, which nonspecifically impair the development of a healthy adaptive immune system. Nevertheless, PRRSV infections weaken piglet growth, general health, and destroy alveolar macrophages (1) that can indirectly result in death from secondary enteric and respiratory pathogens. Indeed, myelomonocytic cells are the primary targets of PRRSV infection, with CD163 serving as the entry receptor. This is the reason why the virus replicates particularly in tissues such as the lung and why i.m. infection probably behaves in the same way as i.n. infection (12). Activated macrophages can circulate throughout the body and effectively redistribute virus to many tissues that do not contain as many CD163^+^ cells, such as the thymus or brain (**Figure 1**; 1). Therefore, the role of macrophages in thymic dysregulation may be crucial, as they can act as a Trojan horse that easily delivers the virus to otherwise inaccessible tissues.

Three MLV strains were used in a mixture because they showed comparable effects *in vitro* when used separately. The reason for the MLV mixture was to conduct initial studies to determine whether at least one of the MLV behaves similarly to wild viruses. In such a setting, it was essential to use both types of wild strains to demonstrate that under the given experimental conditions, the age and condition of the animals and the same experimental design, dysregulation by both types of wild strains occurs. Without evidence that at least one of the MLV is unsafe, it would be impossible to conduct further studies. It is now clear that future studies are needed to (1) test how old pigs need to be to be resistant to immune dysregulation and (2) test whether some individual MLV (of these three but even others) never cause immune dysregulation. While we are working on point 1, it is very difficult to start with point 2. To reliably and conclusively demonstrate the degree of safety and unequivocal exclusion of a possible thymic dysregulation, one would need large cohorts of piglets of different ages with multiple replicates for each MLV. Such trials are more of a challenge for the pharmaceutical companies producing current and any future MLV.

The use of the MLV mixture can be criticized because of possible interference or recombination between MLV strains, as viremia and transmissibility may increase (49). Recombination can occur as early as 7 dpi and is common later (50). Therefore, it is highly likely that mixed inoculation of MLV strains generates recombinant virus. On the other hand, MLV vaccination is often used to control previous PRRSV infections, and recombination must occur regularly. In such case, the mixed MLV strains are more similar to what happens in the field. In addition, the results of this study for the MLV mixture are comparable to wild-type viruses that were used separately and cannot recombine. If immune dysregulation were due to recombination, it would not be observed in separate strain infections, which was not the case here or in previous studies (1).

Different degrees of thymic architecture destruction were observed in individual animals. We highlighted this feature for VACC (compare **Figures 4E**, **F**), but it was also observed for wild strains. Our explanation for mild or severe thymic destruction comes from previous studies, in which the onset of thymic destruction was transient and manifested differently between 7 and 20 dpi (1). If the progression is different in different piglets, the analyses may also be different if performed in a fixed time period. On the other hand, the variations in thymic destruction could also be related to possible mutations or recombinations of the virus mentioned earlier. We consider this possibility less likely because the genotypes of PRRSV type 1 and type 2 have only about 60% nucleotide sequence identity, but both variants cause thymic dysregulation. In any case, the relationship between the extent of thymic destruction and the genetic variants of the virus needs further investigation, and until this issue is resolved, MLV should be considered potentially dangerous.

## 5 Conclusion

MLV can behave comparably to wild PRRSV strains in causing immune system dysregulation in young piglets. The strategy of using MLV in older sows may seem reasonable knowing that MLV can be efficiently distributed throughout the body and infect fetuses (51). Similarly, MLV can hide in tissues and survive in mothers (51) who are raising their very young offspring.

## Data availability statement

The original contributions presented in the study are included in the article/**Supplementary Material**. Further inquiries can be directed to the corresponding author.

## Ethics statement

The animal study was approved by Expert Commission for Animal Welfare of the Ministry of Agriculture of the Czech Republic. The study was conducted in accordance with the local legislation and institutional requirements.

## Author contributions

KS: Conceptualization, Data curation, Formal analysis, Investigation, Methodology, Writing – review & editing, Validation. MT: Conceptualization, Funding acquisition, Investigation, Methodology, Project administration, Supervision, Writing – review & editing. JSi: Conceptualization, Formal analysis, Investigation, Methodology, Project administration, Writing – original draft, Writing – review & editing. SS: Investigation, Methodology, Writing – review & editing. SP: Investigation, Methodology, Writing – review & editing. HKS: Data curation, Formal analysis, Investigation, Methodology, Validation, Writing – review & editing. SA: Data curation, Formal analysis, Investigation, Methodology, Writing – review & editing. RM: Data curation, Formal analysis, Investigation, Methodology, Writing – review & editing. JSa: Data curation, Formal analysis, Investigation, Methodology, Writing – review & editing. HS: Data curation, Investigation, Methodology, Writing – review & editing. KN: Conceptualization, Investigation, Methodology, Writing – review & editing. LL: Formal analysis, Investigation, Methodology, Writing – review & editing. PH: Investigation, Methodology, Writing – review & editing. MK: Investigation, Methodology, Writing – review & editing. BD: Investigation, Methodology, Writing – review & editing. MSJ: Investigation, Methodology, Writing – review & editing. VH: Data curation, Formal analysis, Investigation, Methodology, Writing – review & editing. TH: Formal analysis, Investigation, Methodology, Writing – review & editing. JB: Conceptualization, Writing – original draft, Writing – review & editing. MS: Conceptualization, Data curation, Formal analysis, Funding acquisition, Investigation, Methodology, Project administration, Resources, Supervision, Validation, Writing – original draft, Writing – review & editing.
