# Supplementary material for: Modified live vaccine strains of porcine reproductive and respiratory syndrome virus cause immune system dysregulation similar to wild strains
Source: Front Immunol. 2024 Jan 12;14:1292381. doi: 10.3389/fimmu.2023.1292381 (PMC10811158; doi:10.3389/fimmu.2023.1292381)
Supplement: Supplementary file 1 [file DataSheet_1.pdf]

Supplementary material 1

A: Flow cytometric gating strategy for T-lineage cells from the thymus

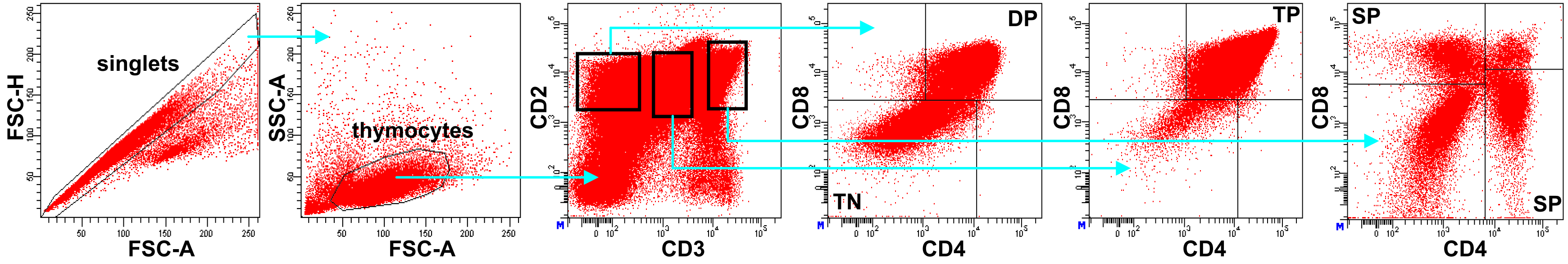

B: Flow cytometric gating strategy for thymic B cells

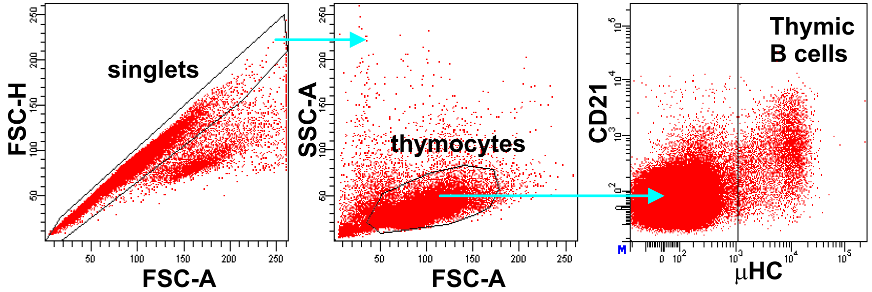

C: Flow cytometric gating strategy for peripheral T cells (BAL as an example)

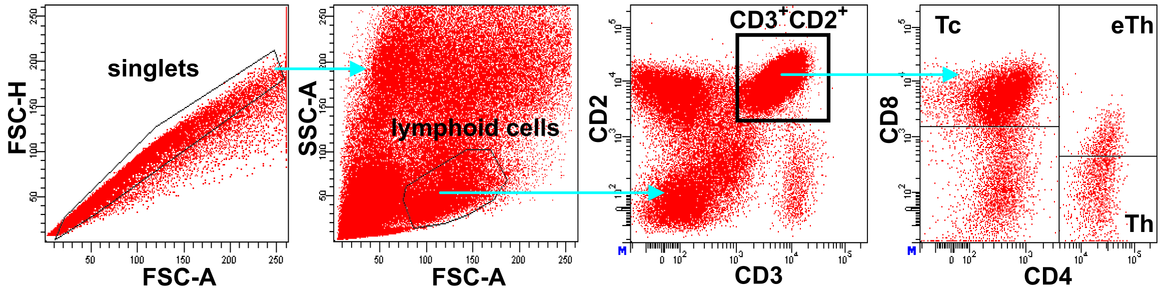

D: Flow cytometric gating strategy for peripheral B cells (BAL as an example)

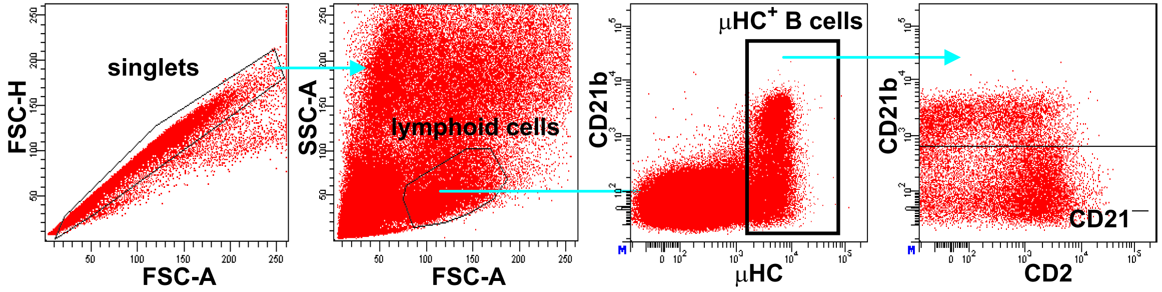

Supplementary material 2

		CTRL	VR2332	Lelystad	VACC
Thymic precursors	TN	GCCAGCAGCTTAGGCGGGACAGGGCAAG<br>GCCAGCAGCTTAGACAGGACACTGCA<br>GCCAGCAGCTTGACAGGGAAATG<br>GCCAGCAGCTTACGAGCCATAG<br>GCCAGCAGCCCACCTTCGGGGACAGATGAT<br>AGCGGTAGACCGAGAG<br>AGCAGTAGAGATGGGAGACAGGGGGGGGGGACGG<br>GCCAGCAGCTACGGAGCTATATAC<br>AGCAGTGGCACGCGGGGGGCCTTACGTA<br>GCCAGCAGCTTGGGGGCTGGGGAA<br>GCCAGCAGCAACCA<br>GCCAGCAGCGAGAAAGAAGGGGTGAGGG<br>ACAACAATAGATCCGGGGGCAGAAAAGCTC<br>GCCAGCAGCGAGGACAGGGGGGGCCAC<br>GCCAGCAGTCCG	AGCAGGAGATCGGGAACGACAGGAGAGG<br>AGCAGGAGATCGGGAACGACAGGAGAGG<br>AGCAGTAGCGACAGCCGGGACAGGGGTTGC<br>AGCAGTAGAGATCTCAGGGGGGT<br>GCCAGCAGTACCGGAGCTATGGGGGATAGG<br>AGCAGTAGAGATAAACAGACCCCAC<br>AGCAGTAGCGTGAGGGAGA<br>AGCACAAAGGGAGACAGGGGACG<br>AGCAGACCGACCGGGGTAG<br>AGCACGACATCCGTG<br>GCCAGCAGCTTAGGAATCCGTCTGAT<br>AGCAGTAGAGGGGGACCGCCA	AGCAGTAGGGGGC<br>GCCAGCAGCTTTGA<br>GCCGGGATGTCCGGGGGGAGG<br>ACCACCGAACAGGGGGGCCAAACCAT<br>AGCACCCCGAGTCTCGACCT<br>AGCAGACAGGGAGGCTCCGTGG<br>GCCAGCAGCTTCGAAATGGAGCTATCA<br>GCCAGCAGCTTAAAGGGATTAG<br>GCCAGCAGCTTATYTAGGATACAAT<br>AGCAGTAGTGGCGGGACAGGCGA<br>AGCAGCCATAGAGA	GCCAGCAGCCCAGTGAAGGGACAGGGGGGGCGAATTCG<br>GCCAGCAGCCTAAGCCCAGGAGCTATTTGGG<br>AGCAGTAGTACGGGGT<br>GCCAGCGGGCTAGGGGACGT<br>AGCAGACAGGCCGC<br>GCCAGCAGCCTTCACGGGACAGCCTA<br>AGCAGTAGAGATCAGGGGGGCGCGC<br>GCCAGCAGGATCAATACGGGACAGCCCAAC<br>GCCAGCAGCTTAGTACGG<br>AGCAGTAGCCTCACGGGGGGGCCCCA<br>AGCAGTCCAACCCTGTTGCCACTGGGG<br>AGCAGTAGGGTCGGGGACCCATGG
	DP	AGCAGTCATATCAGGGG<br>GCCAGCAGCACCTGACAGGCTCAGGG<br>AGCAGCCCCGCCAGGGG<br>GCCAGCAGCTTAGTGACCCAGGGAAAAGCGGTG<br>AGTAGTCTAACCGGGCCCGAGTACG<br>GCCAGCAGCCACGGGGGCTATGGCGGG<br>ACGTGGCTATGGGGGCCGA<br>AGCAGTAGCACTTCGAGGGTCGG<br>AGCAACTCCCGGGCCACAGG<br>AGCAGTTCAGGGT<br>GCCAGCAGCTTGAACACGGACAGC<br>GCCAGCAGCTTAAGGGGCAGGGGA<br>AGCAACAGGGGGGGC<br>GCCAGCAGCTTGGGACAAA<br>GCCAGCAAGACCAGGAGCTATGGGGGGCCGGC<br>GCCAGCAGCCCCGGGACAGGGGGGGC<br>GCCAGCGGAAGAGGGGGCAGCAGC<br>AGCTCCCATGAGGGGG<br>AGCAGTAGAGATCTTATA	AGCAGTAGAGATCGCGGACACA<br>AGCAGTAGAGATCGCGGACACA<br>GCCAGCAGCT<br>AGCAGTAGAGTTAGGGATGGACAGGGGGACTG<br>GGAACCCGGAGCTCCCAAG<br>GCCAGCAGCTACGGCGGAGTT<br>AGCAGTAACCTAAAGGTGA<br>GCCAGCGGTCAG<br>AGCAGTAGAGATCCCACGGGGGGCTGG<br>GCCAGCAGCTTGGGACAGGGG<br>AGCAGTAGAGAGAGGGACAAGG	AGCAGTCCCGGGGGGCCTAT<br>GCCAGCAGCTTAGCCCGGGACAGGGGGG<br>GCCAGCAGCTTAGGAGGG<br>GCCAGCAGCTTTGAGCT<br>AGCAGTGGAGAGCTACGGGGGGGGGGT<br>AGCAGTAGAAGCTACGGGGGGAGACA<br>GCCAGCAGGACAGGAGAGGGCC<br>AGCAGCCCCACAGGTTCCCAAGGCC<br>GCCAGCAGCTTTCGGACAGG<br>AGCAGTAGCCCTGTAAGGGGCCGGG<br>AGCAGTAGTGGGACAGGGAGG<br>AGCAACGAGGGACATG<br>AGCAGTAGAGTTGGGACTTTCA	AGCAGTAGAATTGCGGGGGGGGG<br>AGCAGTAGAATTGTGGGGGGGGG<br>AGCAGACAGGATCA<br>AGCAGTCCTACAGGGGAG<br>AGCGGGGAGGTAG<br>AGCAGTGTTGGGGGGGGCGATA<br>GCCAGCAGCCCCAGTGAGCAATGGGGGGGAGAACTA<br>AGCAGTAGACTCGGCTATGGGGGGGCCTCCCCG<br>AGCAGTAGGAAAGGGACAGGGGGGGCCGA<br>GCCAGCAGCACCTTAGA<br>ACCAAGACGACTGAAGG
	TP	AGCAGTAGAGATTTATTGG<br>GCCAGCCGGGGGGTAAAAA<br>AGCAGTAGAGTAGGGGGATC<br>GCCAGCAGCGTCCGACAGGGTTCG<br>GCCAGCAGCTTAGGGGGACAGTTT<br>GCCAGCAGCTCGGGACCCCT<br>AGCAGTAGTCAGGGACAGGG<br>GCCAGCAGCTTAGGCAGGAGC<br>GCCAGCAGCTTCGGGAGCTATGGGGGGGG<br>GCCAGCAGCTCACTGGGAGCTATGGGGG<br>AGCAGTAGTAACAACAGGGGCCATGG<br>GCCAGCAGCTGGGACAGGGGG<br>GCCAGCAGCTCCCAAACGGGGGCAGGTGTCC	AGCAGTAGAGATTCGGCGCTATT<br>AGCAGTAGAGATTCGGCGCTATT<br>AGCAGTAGCGCACGCGCTGAGCA<br>AGCAGTAGGGGAGCTGAGCA<br>GCCAGCTTCCTGGGGGACGTCTACGCTGAGCA<br>AGCTCATCGGCTATCCTACAAG<br>AGCAGTAGGGGGGGGTGGG<br>AGCAGTCCGGGGG<br>GCCTCCTCCTTCGGGGG<br>AGCAGTAGAGTCCCACGAAGTAGTTGG<br>AGCAAA<br>GCCAGCAGCTTAGGGGGGCT<br>AGCAGTAGAAGCTATCTGG<br>GCCAGCAGCTTAGTAGGGGGGGGGGGGGTCAG<br>AGCAGTAGGGGAATTGGA<br>AGCAGTTTGTATCCACAGGGGGGAGTGGGGAGAAG<br>GCCAGCAGCCGGGACAGGGT<br>GCCAGCAGCTTGCAGGGGGGAAGGTGG	GCCAGCAGCCCTCAGACGGGAT<br>GCCAGCAGCCCTCAGACGGGAT<br>GCCAGCAGCTTATATTTTGAGG<br>GCCAGCAGGGGGGGCAACACG<br>AGCAGTAGAGACAGGGGGA<br>GCCAGCAGCCGGGACAGG<br>AGCAGCTAT<br>GCCAGCAGCGGACAAT<br><br><br>AGCAGTAGAGATGGGCAC<br>GCCAGCAGCTTACAGGGAG<br>AGCAGTCCCGGATAC<br>GCCAGCAGCTTGTTCGA	AGCAGCAGGGATAGA<br>GCCAGCAGCTTAGGGGCAGGGGGGGCGA<br>ACCCGGGGACAGGGAATA<br>GCCAGCAGCTTTGCCGGGGGGGGGG<br>GCCAGCAGCTTACGTGGACAG<br>GCCAGCAGCGCTAGGACAGGGAC<br>AGCAGTAGAGATGTTCACGGGCTGGGAT<br>AGCAGTAGCATACAGGGCAGGGGT<br>GCCAGCAGCTTAGTGACAGGGGGG<br>ATCCCCCGGGACAGA<br>GCCAGCAGCTTAGTGGGGACAGGCCC
$CD4^+$ Th cells	Thymus	GCCAGCAGCTCCCCGGGACAGGGGGGACCT<br>AGCAGCCGGGGGGGGGTGACG<br>GCCAGCATCGGAGCCCTAGGCTTTGAGCA<br>AGCAGTAT<br>GCCAGCGGAAGAAGCT<br>GCCAGCAGCTTACATTTGAGCTATGGGTCC<br>GCCAGCAGCCAACTGGACAGGG<br>AGCAGTTTGGAATTTGGAGACACCTACC<br>AGCAGTGAGCTCCACCTCGGGG<br>AGCAGTATCCTCGGACAGGGGGTA<br>GGGGGGGG<br>GCCAGCCGACCTATGTTG<br>GCCAGCAGTTTTGACGGGACACTC<br>AGCAGTAAGGGCCAGGGAAA<br>GCCAGCAGCCCCCTATGGGGGG	AGCACTTTTGAGG<br>AGCACTTTTGAGG<br>GCCAGCAGCTCGATGGTGTTAGGGGCCACCCTGACCTTTGGGG<br>GCCAGCAGCCCGGGGAGCCA<br>GCCAGCAGCCCCACGTCGAGGGCACGCGTG<br>GCCAGCAGCTTAGCGGGGG<br>GCCAGTCTCACAGGATCTTTGGGG<br>GCCAGCAGCCTCGGACAGGGCGGGG<br>AGCAGTAGAGACCTGGGGCGCTC<br>GCCACAGGAAACCGGGA<br>AGCGCCCTCAGGACAGGGGACCA<br>AGCAGTAGAGATGGACAGGATTTTACGG<br>GCCAGCTTCCCCGGG<br>AGCCAAGAAAGACACAGGG	GCCAGCAGCTTATGGACAGC<br>GCCAGCAGCCCCGAGCTAGGGG<br>AGCAGTAAGGACAGGGTGGG<br>GCCAGCGACAC<br>GCCAGCAGCGTACCGGGACAGAATAA<br>GCCAGCAAACGGTGGCTATGGGGGGGCCGAC<br>GCCAGCAGCTTATATGGCCGGAGCCCCAGG<br>AGCAGTAGAAAAACCGGGACAGGG<br>GCCAGCACCGATAGTCTTAGGACAGGCGAA<br>AGCGACAGGGGGGGAG<br>AGCAGCCTCGGACAGGG<br>AGTACTAATGGGGGTG<br>AGCAGTAGAGATCGGCAGAGA<br>GCCAGCAGCGGGGGGCCCAAGCGG	AGCCGGAGGGTTGGG<br>GCCAGCAGCTTAGTAAATGGGGGGGGG<br>GCCAGCACAGGACCGGCTC<br>AGCAGAGACCCTGGCCGTG<br>AGCATGCGGACAGGCTTCA<br>GCCAGCAGCCGGGACTCGT<br>GCCAGCAGCTCCGGGACAGGGGGGGG<br>GCCAGCAGCCCCAAAGG<br>GCCAGCAGCGGAGGGGGGGCAGACACTGAAGTC<br>AGCAGACGTTCGGGACA<br>AGCAGTCCCCCCCACGG<br>AGCAGTTTCGACC<br>GCCAGCAGCTTAGGGACGTCCTT<br>AGCAGTAGAGAACTCGGGAGG<br>GCCAGCAGCGGACAGGGGGGGC
	Spleen	AGCAGTAGTCCGGACAGGA<br>GCCAGCAGCATGGGGACAGGGGGGGAG<br>AGCAGTAGAGTGGACAGGGGGGTC<br>GCCAGCAGCCCATTGAGGGGTAAA<br>GCCAGCAGCTTAAAGGAGA<br>GCCAGCAGCTCGGACAG<br>GCCAGCAGCTTAGTAAAGGG<br>GCCAGCAGCCAGGGGG<br>AGCAGTAGGGGGGACAGGGTCAA<br>CAGCAGTATTATGGGGAGGG<br>AGCATGGCAGGGGGGACAT<br>AGCAGTGGAGACTGGC<br>GCCAGCAGCTTGGGCAGGGGG<br>AGCAGTATTATGGGGAGGG<br>GCCAGCAGTAGAAACAGGGGGG<br>GCCAGCAGTCTGTTCGAGACAGAG<br>GCCAGCAGCTTACAGGAAGGG<br>GCCAGCGCCTGGGGGGGGAA	AGCAGTTCTTCCGGAGCTAT<br>GCCAGCAGCTTAGGGACAGG<br>GCCAGCAGCTACAGGGGGGAAG<br>GCCAGCAGCTGGAGCT<br>GCCAGCAATGAGGACAGGGGTCAGGAGCGGTAT<br>GCCAGCAGTCCCGGGACGCGG<br>GCCAGCAGCTTAGA<br>GCCAGCAGTCCGATATGGGGGGGTCCTAGGTTGA	GCCAGCAGCCCTCAGACGGGAT<br>GCCAGCAGCCCGACAGTTG<br>GCCAGCAGCCCGACAGTTG<br>AGCAGACGACAGGGGGTGGT<br>GCCAGCAGCTTAACT<br>GCCAGCAGCCGAGCTACGGGGGGGGGG<br>GCCAGCAGCCCGGGACAGGGGGT<br>GCCAGCAGCTTAGACAGGTTT<br>GGACAGGCATGC<br>GCCAGCAGCTTGGGGGGGGGGGGCCT<br>GCCAGCAGCTCGACCCGGGTCC<br>AGCAGTAGGACAGGGTT<br>AGCAGGGACAGCTTTGGAGACACCTACTTT<br>AGCAGTAGAGGGGACAGG	GCCAGCACGACCATGGA<br>GCCAGCACGACCATGGA<br>AGCAGTTTAATGGGGGGTCGTG<br>GCCAGCAGCTTGACCGGATTAGAG<br>GCCAGCAGCCTAAATAGTTT<br>GCCAGCAGCCATGGGGGGAGAA<br>GCCAGCAGCTTACAGGGGGGCGA<br>AGCAGTACTTGGACCACC<br>AGCAGTAGAAGG<br>AGCAGTAAGTCCGGG<br>AGCAGTAGAGCCGGACAGGCCCC<br>AGCAGTAGAGATCGTAGTATTTTGGTGT<br>GCCAGCAGCTGGGGCAG<br>AGCCAACCGGGACAGGT
	BAL	GCCAGCTGGGGTC<br>GCCAGCAGCTTGGCGTAC<br>GCCAGCAGCCGGGAC<br>GCCAGCAGCTCGGGACAGGGGGG<br>GCCAGCAGACGGACAG<br>GCCAGCAGCCGGGACAGGGGGGG<br>GCCAGCAGCGGGGGGGGGCGCTTA<br>GCCAGCAGCTTAGTCG<br>GCCAGCAGCAACAGGGG<br>GCCAGCAGCTTAATGGACAGGGGGC<br>GCCAGCAGCTTAGGAA<br>GCCAGCAGCTCTGTAG<br>GCCAGCAGCTCGGTGGGGGGGT<br>GCCAGCGGACGAA	GCCAGCTTCCGGGACAGGGG<br>GCCAGCTTCCGGGACAGGGG<br>GCCAGCTTCCGGGACAGGGG<br>GCCAGCTTCCGGGACAGGGG<br>GCCAGCTTCCGGGACAGGGG<br>GCCAGCTTCCGGGACAGGGG<br>GCCAGCTTCCGGGACAGGGG<br>GCCAGCAGCTTAGCCTTGACCCCGGATG<br>GCCAGCAGCTTAGCCTTGACCCCGGATG<br>GCCAGCAGCTTAGGGGGACAGGGGTTGAA<br>GCCAGCAGCTTAGGGGGACAGGGGTTGAA<br>AGCAGTAGTATGGCTATGGGG<br>AGCAGTAGAGCGGGACAGGT	AGCAACAGGGG<br>GCCAGCAGCTTAGGACAGGGGGG<br>GCCAGCAGCTATCGACAGGTTCGTTCGAG<br>AGCAGTAAAG<br>AGCAGTAGAGGG<br>AGCAGTAGACAGGGGG<br>GCCAGCAGCTTGTGG<br>GCCAGCAGCTCGGGCAGGAG<br>AGCAGTAGACAG<br>GCCAGCAGCTTAAGGGGG<br>AGCAGTGGACAGGGG<br>GCCAGCAGGGGCCTT<br>AGCAGTAGGACGGA<br>AGCAGTAGAGAGGGGGGGGGGAATT	GCCAGCAGCTCCAGGGGGGGCGC<br>GCCAGCAGCTCCAGGGGGGGCGC<br>GCCAGCAGCTCCAGGGGGGGCGC<br>AGCAGTAAAAGGGACCTCA<br>AGCAGTAAAAGGGACCTCA<br>AGCAGTAGGAGGGGGGCGCT<br>AGCAGTAGGAGGGGGGCGCT<br>AGCAGTAGGAGGGGGGCGCT<br>AGCAGTAGGAGGGGGGCGCT<br>AGCAGTAGGAGGGGGGCGCT<br>GCCAGCAGAACAGGTTAT<br>GCCAGCAGCCCTCGGCAGGT<br>AGCACTCGGACAGGAA<br>GCCAGCAGCTTATTGGGACAGGG<br>AGCAGTAGAGGTGGAGCTATGGGGTCCATG
$CD8^+$ Tc cells	Thymus	GCCAGCAGTGTAGCGACGGAAGG<br>GCCAGCAGCTTAGCAAGGCTC<br>AGCAGTAGAA<br>GCCAGCAGCTTCAA<br>GCCAGCTTAT<br>AGCAGTAGAGACAGGGTAC<br>GCCAGCAGCGTCGAAGTCCCC<br>AGCAGTAGGA<br>GCCAGCAGCCGGGACAGGGGG<br>GCCAGCAGCTTAGTTTCTC<br>AGCAGTAGAGAGGGGAGG<br>AGCAGAGCTCTTT<br>AGCAGTAGAAATTTCGCACCGGGTCGT<br>AGCAGTAATCGGGACAA<br>AGCAGAGGTGGG<br>AGCAGCCTAAGATCTGGACAGGGCGG<br>AGCAGTAGGGGGGCCGC	AGCAGTAGGACA<br>AGCAGTAGGACA<br>AGCAGTGGTC<br>GCCAGCAGCTTTAGGGTA<br>AGCAGTGGAAGG<br>GCCAGCCAATCCGGGCAGTGGG<br>AGCAGTAGAGATGAAGGGGGGGTACG<br>GCCAGCAGCGACAGGGGGGCCGCCTA<br>AGCAGTAGGGGGGGCG<br>AGCAGTAGTAAACAGGGGGTG<br>AGCAGGACAGG<br>GCCAGCAGCCCTAGGACAGGGGGGGCGCGGG<br>AGCAGACAGGG<br>AGCAGTCCACGGAGCTATGGGTACGCTGAGCA<br>GCCAACAAATCCGGGGACAGGGCAAATTCGCCCCTCC<br>AGCTCCAGGGTGGGGA<br>AGCAGTAGACAGGTT	GCCAGCAGCTTAGAATGGGGGC<br>AGCAGCTTGGGGGGGGGCCT<br>AGCAGCTTGGGGGGGGGCCT<br>AGCAGCTTGGGGGGGGGCCT<br>AGCAGCTTGGGGGGGGGCCT<br>AGCAGCTTGGGGGGGGGCCT<br>GCCAGCAGCGGGACAGGCACTGG<br>AGCCGCATAGAGCTATGGGGCGC<br>GCCAGCAGCACAGGGGG<br>GCCAGCAGCGGGACAGGG	AGATTCGGG<br>AGATTCGGG<br>AGATTCGGG<br>AGATTCGGG<br>AGATTCGGG<br>AGATTCGGG<br>AGATTCGGG<br>AGATTCGGG<br>AGATTCGGG<br>GCCAGCAGCTTAGGACAGA<br>GCCAGCAGCTTAGGACAGA<br>AGCAGAAAGACGGGGGACAGGGGGAGG<br>AGCAGCGAAGATCTTCGGGGA<br>GCCAGCGCCATGCCCGGGACAGGGGG<br>GCCAGCAGCTTAGTAAAGGT<br>AGCAGTCGACGGGGGGGGGGTTTAAAA<br>GCCAGCAGCTTGGAGGGGGGCC
	Spleen	AGCAGTTAGGACAG<br>AGCAGCACGGGCAGGGACA<br>AGCAGTTACGGG<br>AGCAGTGGCAGGGGGGGT<br>GCCAGCAGCTTAGTACTGGTGGGGGGGCA<br>AGCATCGGGACAGGG<br>AGCAGGGGACT<br>AGCAGTGGTCAGGCCAG<br>GCCAGCAGCTCAGGCCAGGCGAGGG<br>GCCAGCAGCTTAGGGCTGTTG<br>AGCATTACCGGGACAGGGGGGG<br>AGCAGTAGCTTTG<br>GCCAGCAGCTCCCCGGGAAGC<br>AGCAGTAGAGATGCCAGGCCT<br>AGCAGTAGGGACAGAG<br>GCCAGCAGCTTGGGCACAGGGC	AGCAGTAGGACA<br>AGCAGTAGGACA<br>AGCAGTAGGACA<br>AGCAGTGGTC<br>AGCAGTGGTC<br>AGCAGTGGTC<br>GCCAGCAGCTTTAGGGTA<br>GCCAGCAGCTTTAGGGTA<br>AGCAGGAT<br>AGCAACCCCGGGACAGGGTGG<br>AGCAGTCTACAATTGGGGACAGGGA<br>GCCAGCAGCTTACGAATGGGGGGGGGG<br>GCCAGCAGCTTATGGCGGGACAG<br>AGCAGTAGAGAGGGGATTAGAC	GCCAGCAGCTTAGAATGGGGGC<br>GCCAGCAGCTTAGAATGGGGGC<br>GCCAGCAGCTTGGGGGGGGGGCCT<br>GCCAGCAGCTTGGGGGGGGGGCCT<br>GCCAGCAGCTTGGGGGGGGGGCCT<br>GCCAGCAGCTTGGGGGGGGGGGGCCT<br>GCCAGCAGCTTGGGGGGGGGGGGCCT<br>GCCAGCAGCCACACGGGGCCAGG<br>GCCAGCAGCTTAGTACAG<br>ACTAGCTTACAATGGGTCC<br>GCCAGCAGCGGACAGGGTGG<br>GCCAGCAGCTTGGGGGGGGGCCT<br>GCCAGCAGGGGGGGC<br>AGCAGTATAAGGGGTCCC<br>GCCAGCAGCTTGGGAC	AGATTCGGG<br>AGATTCGGG<br>AGATTCGGG<br>AGATTCGGG<br>AGATTCGGG<br>AGATTCGGG<br>AGATTCGGG<br>GCCAGCAGCTTAGGACAGA<br>GCCAGCAGCCCCCCTTTTT<br>GCCAGCAGCCCCCCTTTTT<br>AGCAGTAGGGTTGGAGGG<br>AGATCCGGG<br>GCCAGCAGTTCGGAGCTATGGGGGGGGC<br>AGCAGTACAGGGG<br>AGCACGGACAGGGG
	BAL	AGCAGGCGGACAGAATCC<br>AGCAGTAAGGGGGGG<br>GCCAGCAGCCAGGG<br>AGCAGTAA<br>AGCAGTGG<br>GCCAGCAGCTTATTAGGGGAGCTA<br>GCCAGCAGCTTAGGTGACAGAAC<br>AGCCGGGTC<br>AGCAGTCCCCCGA<br>AGCAGTAGGGACAGGGGGGG<br>AGCAGTAGAGGGACAGGGT<br>AGCAGTAAGGACAGGGG<br>AGCAGTACCCGGACGGGGCAG<br>AGCAGACAG<br>GCCAGCAGCTTAAGG<br>GCCAGCAGCTGGACAGGGGAGA<br>GCCAGCAGCTTGAAGCTA	AGCAGTAGGACA<br>AGCAGTAGGACA<br>AGCAGTAGGACA<br>AGCAGTAGGACA<br>AGCAGTGGTC<br>GCCAGCAGCTTAAGTTTTGGGGGGCAA<br>GCCAGCAGCTTAAGTTTTGGGGGGCAA<br>GCCAGCAGCTTAAGTTTTGGGGGGCAA<br>GCCAGCAGCTTAAGCTTTGGGGGGCAA<br>GCCAGCAGCTTAAGCTTTGGGGGGCAA<br>AGCAGGAT	GCCAGCAGCTTAGAATGGGGGC<br>GCCAGCAGCTTAC<br>AGCAGTAGGGA<br>AGCAGTACAGGGGGG<br>GCCAGCACCCTTGGACAGACT<br>AGCAGTAGAATAGGGGG<br>AGCAGCATGGAC<br>GCCAGCAGCACAGGG<br>AGCAGTAGAGAATTGAA<br>GCCAGCAGCGACAGGTG<br>GCCAGCACCCTACCGGG<br>GCCAGCAGCTTAGGGGGGAAA<br>GCCAGCAGCTTAGGG<br>AGCAGTAGAGACAGGG	AGATTCGGG<br>AGATTCGGG<br>AGATTCGGG<br>AGATTCGGG<br>AGATTCGGG<br>AGATTCGGG<br>AGATTCGGG<br>AGATTCGGG<br>AGATTCGGG<br>AGATTCGGG<br>AGATTCGGG<br>AGATTCGGG<br>GCCAGCAGCCCCCCTTTTT<br>AGCAGTAGGGTTGGAGGG<br>GGGGGGGGACAGGGA
